# Effect of long term inhaled corticosteroid therapy on adrenal suppression, growth and bone health in children with asthma

**DOI:** 10.1186/s12887-019-1760-8

**Published:** 2019-11-05

**Authors:** Anuradha KWDA, Prematilake GLDC, Batuwita BAUI, Kannangoda KASR, Hewagamage US, Wijeratne S, Lankatilake Kantha, de Silva KSH

**Affiliations:** 1 Lecturer Faculty of Medicine, University of Colombo, Colombo, Sri Lanka; 2 Acting paediatric pulmonologist, University paediatric unit, Lady Ridgeway Hospital for children, Colombo, Sri Lanka; 3 Acting paediatric endocrinologist, Lady Ridgeway Hospital for children, Colombo, Sri Lanka; 4 Research Assistant, University paediatrc unit, Lady Ridgeway Hospital for Children, Colombo, Sri Lanka; 5 Medical Officer, National Hospital of Sri Lanka, Colombo, Sri Lanka; 6 Medical Officer, Lady Ridgeway Hospital for Children, Colombo, Sri Lanka; 7 Laboratory Director, Vindana Reproductive Health Centre, Colombo, Sri Lanka; 8 Associate Professor, Department of Community Medicine, Faculty of Medicine, University of Colombo, Colombo, Sri Lanka; 9 Professor in paeditrics ,Faculty of Medicine, University of Colombo and Lady Ridgeway Hospital for Children, Colombo, Sri Lanka

**Keywords:** Asthma, Inhaled corticosteroids, Growth, Adrenal suppression, Bone metabolism, Vitamin D

## Abstract

**Background:**

Inhaled corticosteroids (ICS) are the most effective treatment for children with persistent asthma. However adverse effects of ICS on Hypothalamo Pituitary Adrenal (HPA) axis, growth and bone metabolism are a concern. Hence the primary objective of this study was to describe the effects of long term inhaled corticosteroid therapy (ICS) on adrenal function, growth and bone health in children with asthma in comparison to an age and sex matched group of children with asthma who were not on long term ICS. Describing the association between the dose of ICS and duration of therapy on the above parameters were secondary objectives.

**Method:**

Seventy children with asthma on ICS and 70 controls were studied. Diagnosis of asthma in selected patients was reviewed according to the criteria laid down by GINA 2018 guidelines. The estimated adult heights were interpreted relative to their Mid Parental Height (MPH) range. Serum calcium, alkaline phosphatase and vitamin D levels were analyzed in both groups and cortisol value at 30 min following a low dose short synacthen test was obtained from the study group. The average daily dose of ICS (Beclamethasone) was categorized as low, medium and high (100–200, 200–400, > 400 μg /day) respectively according to published literature.

**Results:**

Heights of all children were within the MPH range. There was no statistically significant difference in the bone profiles and vitamin D levels between the two groups (Ca: *p* = 0.554, vitamin D: *p* = 0.187) but vitamin D levels were insufficient (< 50 nmol/l) in 34% of cases and 41% of controls. Suppressed cortisol levels were seen in 24%. Doses of ICS were low, medium and high in 56, 32 and 12% of children respectively. The association between adrenal suppression with longer duration of therapy (*p* < 0.01) and with increasing dose of ICS (*p* < 0.001) were statistically significant.

**Conclusion:**

ICS had no impact on the growth and bone profiles but its dose and duration were significantly associated with adrenal suppression.

## Background

Asthma is a commonly encountered chronic inflammatory disease in children where there is an airway hyper responsiveness leading to obstruction and limitation of the airflow. It is clinically defined by the presence of recurrent respiratory symptoms such as cough, wheeze, shortness of breath and chest tightness that vary over time and in intensity with variable expiratory airflow limitation [1].

Inhaled corticosteroids (ICS) are considered to be the most effective treatment for management of persistent asthma as they prevent exacerbations, improve lung function and the quality of life and reduce hospitalizations and asthma related mortality [2]. Although the side effects of ICSs are less frequent and severe than that of oral corticosteroids, safety concerns with these agents still remain especially in relation to high doses [3]. Effects of ICS on growth of children, bone health and adrenal function are the key areas of concern in studies found in the literature.

Possible suppression of the Hypothalamo-Pituitary Adrenal (HPA) axis leading to adrenal suppression (AS) is an under recognized, life threatening adverse effect [4, 5]. It is a proven complication of most forms of glucocorticoid therapy that persist even after one year of cessation of therapy [6]. Therefore screening of patients for AS is important. This is dependent on many factors such as the type of corticosteroid, duration of therapy and the dose which is proportional to the degree of suppression.

Corticosteroid induced enhancement of osteoclastic activity and suppression of osteoblasts leading to bone resorption is presumed to affect growth and bone metabolism [7, 8].

## Objectives

The primary objective was to describe the effect of long term inhaled corticosteroid therapy (ICT) on adrenal suppression, growth and bone health in children with Asthma.

Secondary objectives were to

- Describe the association between the dose of inhaled corticosteroids and duration of therapy on adrenal functions, growth and bone health in children with asthma
- Compare the growth and effect on the biochemical parameters associated with bone health between children with asthma who are on ICS and age and sex matched group of children with asthma who were not on long term ICT.
- Estimate and compare vitamin D levels in both groups of children and assess the impact of ICS on Vitamin D levels.

## Method

Children aged 3 to 9 years with asthma on long term inhaled corticosteroids for at least 6 months were studied with an age and sex matched comparison group of children with asthma who were not on ICS. A cross sectional descriptive study was conducted at the Asthma Clinic of the University Paediatric Unit of Lady Ridgeway Hospital (LRH), Colombo, Sri Lanka from September 2016 to April 2018.Children older than 9 years were excluded to avoid the pre-adolescent physiological acceleration of growth seen at this age. Because of the possibility of patients with bronchiolitis being included and as nutrition was the main determinant of growth, children under 3 years were not included in the study. Patients who had been on medication for asthma during the week preceding the study were excluded due to the possibility of being given oral steroids which may have interfered with the evaluation of the adrenal axis. Those having long standing illnesses with possible impact on the parameters being evaluated were also excluded from the study. The objectives of this study being to assess the possible adverse effects of ICS related to its dose and duration, the severity and the control of asthma in selected children was not considered in their enrollment.

A convenient sample of 70 children each in the two groups, were studied. Ethical clearance was obtained from the Ethics Review Committees of the Faculty of Medicine, University of Colombo and Lady Ridgeway Hospital, Colombo. Written informed consent was obtained prior to the study, from the mother/guardian and assent when appropriate from the patients.

Socio-demographic and treatment details were recorded using an interviewer administered questionnaire. Heights of the participants were measured using a stadiometer to the nearest 0.1 cm according to the stipulated recommendations. The heights of both parents were also recorded. The mid parental height or target height (TH) was calculated for each patient using the formula given below [9].

Boy = mother’s height + father’s height + 13.0 cm ÷ 2.

Girl = mother’s height + father’s height – 13.0 cm ÷ 2.

Mid parental height range = TH ± 8.5 cm.

The mid parental height range was plotted on the percentile chart at the point of 18 years in each patient. The current height of each child were projected up to 18 years (which is assumed to be the adult height [9] and recorded in relation to the MPH range.

Two milliliters of venous blood was drawn after 6 h of fasting at 8 am from both the study sample and control group, for Serum Calcium, Phosphate, Alkaline Phosphatase (ALP) and Vitamin D. The assay included serum cortisol in the study sample.

Evidence of adrenal suppression was assessed in the study sample by the low dose short Synacthen test. After taking blood for the biochemical analyses, 1 microgram of Synacthen (soluble ACTH) was administered intravenously and a blood sample was taken 30 min later for serum cortisol to assess the function of HPA axis. Peak Cortisol level > 500 nmol/L or 200 nmol/L increment from the basal cortisol level was considered as the normal response [10]. Relationship of adrenal suppression to the dose of ICS was analyzed with the age specific categorization of the cumulative dose of ICS received by children in to high, medium and low dose according to GINA guidelines [1]. Evaluation software, SPSS version 20 was used for data analysis.

## Results

### Adrenal suppression

A peak cortisol level of < 500 nmol/L 30 min after the low dose adreno-corticotropin test, indicating adrenal suppression and therefore an effect on the HPA,was seen in 17 (24.3%) of the study population. ICS had been used for more than 24 months in 13 (76.5%) of the 17 patients in whom adrenal suppression was documented (Table 1).

**Table 1:** Relationship between ICS duration and adrenal suppression (*n* = 70)

Duration of ICS therapy (months)	Adrenal suppression		Total
	Not suppressed	Suppressed	
< 12	9	0	9
12–18	4	1	5
18–24	0	3	3
> 24	40	13	53
Total	53 (75.7%)	17 (24.3%)	70 (100%)

An increase in adrenal suppression was noted with the increasing duration of therapy. This association between duration of corticosteroid therapy and adrenal suppression was found to be statistically significant. (χ^2^ = 12.291; df = 3; *p* < 0.01).

Ten (58.8%) of the 17 patients with adrenal suppression had received high doses of ICS (Table 2). The association between the dose of inhaled corticosteroid therapy and adrenal suppression was statistically significant (χ2 = 29.80; df = 2; p < 0.01).

**Table 2:** Relationship between ICS dose and adrenal suppression (n = 70)

Dose of ICS (μg/day)	Adrenal Suppression		Total
	Not suppressed	Suppressed	
High (> 400 μg/day)	2	10	12
Medium (200–400 μg/day)	23	6	29
Low (100-200 μg/day)	28	1	29
Total	53	17	70

### Height

Effects of long term corticosteroids on the stature of children was assessed by comparing the heights of the children in the two groups in relation to their mid parental height (MPH) range.

No child’s height was below the MPH in either of the groups (Table 3). The association between use of long term inhaled corticosteroids and growth in terms of height was not statistically significant (χ2 = 0.785; df = 1; *p* = 0.376).

**Table 3:** Heights of children relative to MPH range (*n* = 140)

Height	Control group	Study Group	Total
Within MPH range	43 (61.4%)	48 (68.6%)	91 (65.0%)
>MPH range	27 (38.6%)	22 (31.4%)	49 (35.0%)
Total	70 (100.0%)	70 (100.0%)	140 (100.0%)

### Bone health

Calcium and ALP values of the two groups of children did not show a normal distribution thus the Independent samples Mann–Whitney U Test was applied to test for the significance of the results.

The difference between the median calcium values of the study and control groups was not statistically significant (*p* = 0.88). Median ALP values of the study and control groups were 225 U/L and 198.5 U/L respectively, and this difference was statistically significant {(*p* < 0.01), (Table 4)}.

**Table 4:** Comparison of Calcium and ALP values in the two groups (n = 140)

	Control group (70)		Study group (70)	
	Median value	IQR (Inter Quartile Range)	Median value	IQR (Inter Quartile Range)
Calcium (mmol/L)	2.32	2.25–2.37	2.35	2.26–2.44
ALP (U/L)	198.5	147.50–233.25	225	208.25–283.00

### ICS and vitamin D

According to Independent samples Mann – Whitney U Test (Table 5), no significant association was found between long term ICT and vitamin D levels (*p* = 0.886).

**Table 5:** Comparison of vitamin D levels in the two groups (n = 140)

	Control group (70)		Study group (70)	
	Median value	IQR (Inter Quartile Range)	Median value	IQR (Inter Quartile Range)
Vitamin D value (nmol/L)	50.5	44.32–67.00	53.75	44.25–65.75
Vitamin D status: Deficient ≤30 nmol/L, Insufficient =30–50 nmol/L, Sufficient ≥50 nmol/L [11]				

Interestingly 34% of the control group and 41% of the study group showed insufficient vitamin D values (≤50 nmol/L) and 11% of the control group and 4% of the study group showed vitamin D deficiency (≤30 nmol/L).

## Discussion

Identifying the potential adverse effects of inhaled corticosteroids on growth, bone metabolism and the HPA axis had been the focus in many reports in international literature where these aspects had been studied separately. Similar studies have not been reported previously from Sri Lanka. The results of this study demonstrated that long term ICS therapy had a significant effect on adrenal suppression but not on bone health and growth.

### ICS and adrenal suppression

The most serious potential complication of ICS is adrenal insufficiency hence prevention and screening of patients for adrenal suppression (AS) is vital [6].

Endogenous cortisol production is suppressed by exogenous glucocorticoids. When exogenous glucocorticoids significantly alter the pattern of normal diurnal variation in cortisol levels adverse consequences are expected [12]. Acute adrenal insufficiency was seen less commonly compared to adrenal suppression shown by laboratory tests [13].

In our study 24.3% of patients on ICS showed a peak cortisol level < 500 nmol/L at 30 min after the low dose ACTH test indicating AS. This was significantly associated with increasing doses and duration of ICS therapy (Tables 1 and 2). High cumulative dose of ICS (> 400 μg/day) had been received by 58.8% of children who showed adrenal suppression and 76.5% had been on treatment for more than 2 years .

In a survey conducted in UK, 33 patients (28 children, 5 adults) were identified to have adrenal crisis. Out of children 23 had presented with symptomatic hypoglycemia and all of them were treated with 500–2000 μg/day ICS. Majority (76%) of the patients were treated for at least a year [14].

A Finnish study had randomly divided 75 children on ICS into three treatment groups according to the different types of corticosteroids used and performed the low dose ACTH test which had demonstrated AS in 23% of the children using moderate doses of inhaled steroids [15].

Zollner et al had reported AS in 35 to 65% of children on ICS ≥500 μg/day for up to 16 weeks after starting therapy but no significant AS had been detected with low doses [16].

### ICS and growth

In this study the doses of ICS used were low (100-200 μg/day), medium (200–400 μg/day) and high (> 400 μg/day) in 41.4, 41.4 and 17.1% of children respectively and there was no significant difference in height in relation to the MPH range between the study and control groups.

A study done on the adverse effects on growth in patients given ICS for more than 1 year had shown a statistically significant effect on pre-pubertal children but not on pubertal school children [17]. A one-year prospective cohort study carried out in 2001on 124 asthmatic children aged 3 to 16 years on ICS for at least 12 months, the z-scores for height for age, weight for age, body mass index and parental target height for current age concluded that there was no significant effect on the height or body weight of children/adolescents using ICS for more than 1 year at the doses recommended for prevention of asthma [18].

Although no formal dose-response studies have been conducted, data from a European study suggests that the effect of ICS on linear growth is dose dependent. A daily dose of 800 μg was found to have a significantly greater growth suppressive effect with a height velocity of 3.6 cm/year compared to a velocity of 4.6 cm/year at a dose of 400 μg/day [18].

Efthimiou J et al (1998) in their systemic review concluded that the doses of inhaled corticosteroids up to 400 μg/day in children have no significant effect on bones and growth in the large majority of patients with asthma [19]. In a study on prepubescent children with mild to moderate persistent asthma, a small but statistically significant group difference in growth velocity was observed between low doses of ICS and low to medium doses of a Beclamethasone equivalent, favoring the use of low-dose ICS [20]. Similarly, when ICS (Beclamethasone diproprionate) was used on a daily dose of 300 to 800 μg, the mean height velocity decreased when the dose was increased and when decreased or stopped, an increase in the mean height velocity was seen without a reduction in the final height. Thus, a careful assessment of height velocity in all children receiving ICS is recommended [21]. MPH range also provides a useful guide to predict the height percentile for children of parents of average stature [22].

The type of drug or the molecule and the aerosol generating device may alter the magnitude of the adverse effects of ICS on children with persistent asthma. Axelsson I et al (2019) in their review has compared different ICS drugs or inhalation devices with regard to their effects on growth velocity and suggest that they may impact the effect size on growth. Least impact on growth was observed with Fluticasone as the ICS and Easy haler as the delivery device.. However the authors suggest further studies before any recommendation is made on selection of the drug or the delivery devices [23] .In our study we made sure that all the children use the same delivery device at the enrollment and 67 children out of 70 who were on ICS were on Beclamethasone which is the available and commonly prescribed molecule at our setup.

### Bone metabolism

Increased osteoclastic activity and suppression of osteoblastic activity of corticosteroids lead to accelerated bone resorption. Corticosteroids also enhance the inactivation of vitamin D by up-regulating 24-hydroxylase activity. It degrades the physiologically active form of vitamin D_3,_ 1,25-dihydroxycholecalceferol by hydroxylation of the side chain thereby increasing the risk of bone resorption. These actions of corticosteroids are important considerations as vitamin D plays a significant role in bone mineralization [6, 7, 24]. Systemic side effects such as dose related reduction in bone density demonstrated by biochemical markers occur with ICS but are less compared to oral steroids [2]. A significant difference in serum calcium and phosphate between the two groups could not be seen but ALP in the study group was higher and the difference was statistically significant (*p* < 0.01).

Bone Mineral Density (BMD) is a sensitive marker of the effects of corticosteroids on bone and can be used to predict the fracture risk [25]. Decreased BMD has been demonstrated with high doses of ICS but not with low to medium doses [26, 27]. A study on 48 prepubertal children aged 5 to 14 years who were on 400 to 2000 μg/day of beclomethasone dipropionate Metered Dose Inhaler (MDI) has shown slow BMD acquisition in children who were on high doses and no effects with low to moderate doses [28]. Similarly, a case control study [29] and a study by the Childhood Asthma Management Programme (CAMP) group did not show a significant effect on bone density with low to medium doses of ICS [30].

Facilities to evaluate BMD were not available at LRH at the time of this study. Therefore serum calcium, phosphate and alkaline phosphatase (ALP) as biochemical markers of bone health were compared in the two groups.

### ICS and vitamin D

The results of this study revealed no significant association between long term inhaled corticosteroid therapy and vitamin D level (*p* = 0.886) but vitamin D deficiency was seen in 13 (18%) and 6(8%) of the control and study groups respectively.

There is growing evidence that vitamin D plays a role in the pathogenesis of allergic diseases and asthma but it is unclear whether supplementation during childhood may improve the outcome. There is evidence of reduction in active vitamin D levels with the use of corticosteroids [6, 7, 22]. Della Giustina A et al in their review had analyzed the possible links between vitamin D and its supplementation in allergic diseases and concluded that the current evidence to support vitamin D supplementation for the prevention or treatment of allergic diseases including asthma in children is insufficient [31].

### Limitations

Being a descriptive cross-sectional study, this study was unable to demonstrate the effects on growth velocity which was a limitation. Serum Parathyroid Hormone levels were not assayed, which would have been useful to arrive at a more accurate interpretation of the biochemical markers, which is another limitation of this study.

### Conclusions and recommendations

This study concludes that inhaled corticosteroids can cause adrenal suppression when administered in high doses (> 400 μg/day) and for longer durations. (> 18 to 24 months). Close follow up of the patients with regular assessment of the growth and asthma control would enable the dose of ICS to be gradually reduced to the lowest possible dose needed for optimum control of symptoms. A significant effect on the growth of the children who were on ICS was not demonstrated from the findings of this study .

The recommendations proposed from this study would be to pay meticulous attention based on the clinical response to treatment and to be vigilant about the possibility of adrenal suppression when asthma is treated in children.

## Data Availability

○ The datasets used for analysis during the current study are available from the corresponding author on request. All the relevant results are given in the manuscript. Can submit to BMC pediatrics if expected to sumbit.

## Abbreviations

ACTH: Adreno Corticotropic Hormone
ALP: Alkaline Phosphatase
AS: Adrenal Suppression
BMD: Bone Mineral Density
HPA axis: Hypothalamo Pituitary Adrenal axis
ICS: Inhaled Cortico Steroids
ICT: Inhaled Corticosteroid Therapy
LRH: Lady Ridgeway Hospital
MDI: Metered Dose Inhaler
MPH: Mid Parental Height
TH: Target Height
